# Music and Emotion—A Case for North Indian Classical Music

**DOI:** 10.3389/fpsyg.2017.02115

**Published:** 2017-12-19

**Authors:** Jeffrey M. Valla, Jacob A. Alappatt, Avantika Mathur, Nandini C. Singh

**Affiliations:** Language Literacy and Music Laboratory, National Brain Research Centre, Manesar, India

**Keywords:** *raga*, emotion, tonic ratio, *thaat*, music

## Abstract

The *ragas* of North Indian Classical Music (NICM) have been historically known to elicit emotions. Recently, Mathur et al. (2015) provided empirical support for these historical assumptions, that distinct *ragas* elicit distinct emotional responses. In this review, we discuss the findings of Mathur et al. (2015) in the context of the structure of NICM. Using, Mathur et al. (2015) as a demonstrative case-in-point, we argue that *ragas* of NICM can be viewed as uniquely designed stimulus tools for investigating the tonal and rhythmic influences on musical emotion.

## Introduction

Music is the art of sound in time, organized to the principles of pitch, rhythm, and harmony (Limb and Braun, 2008). An important function of music is its capacity to communicate emotions (Tanner and Budd, 1985), a view that has been agreed upon by both music performers (Laukka, 2004) and music listeners (Juslin and Laukka, 2004). Indeed, almost all known forms of music have been recognized for their affective emotional qualities (Goldstein, 1980). However, the exact causal mechanisms by which musical sounds generate emotions are still unclear. Current models posit that specific acoustic factors embedded in a music signal exploit the physical environment, the cognitive and perceptual processing systems, and the structure of the auditory system, to generate emotional responses (Huron, 2006; Thompson and Schellenberg, 2006).

Though the link between music and emotion has been empirically established (Juslin and Sloboda, 2011), most findings lack generalizability across multi-cultural representations of music. Consequently, while music and emotion studies have standardized the use of Western Classical music as a staple source of stimuli, only a handful have incorporated genres of music native to other cultures. This not only precludes interpretations of universality in musical emotions from their findings, it also overlooks musical stimuli which might have advantages as tools for studying musical emotion (Thompson and Balkwill, 2010). The goal of this review is to make such a case for the unique experimental utility offered by North Indian Classical Music (NICM).

In particular, this review will highlight and expand upon the findings of Mathur et al. (2015), to demonstrate that NICM comprises of stimuli that not only permit the study of music and emotional response, but are also uniquely designed stimulus tools to investigate how specific psychophysical features like tonality and rhythm modulate musical emotion as separable factors.

North Indian Classical music (NICM), or *Hindustani music*, is an ancient musical form of India that emerged from a cultural synthesis of the Vedic chant tradition and traditional Persian music (Kaufmann, 1965). The central notion in this system of music are ***ragas***, which are described as musical compositions capable of inducing specific moods or emotions. Past studies have investigated *ragas* and have shown that distinct ragas elicit distinct emotions (Balkwill and Thompson, 1999; Chordia et al., 2008; Wieczorkowska et al., 2010). In a study published recently, Mathur et al. (2015) exploited a novel feature of *raga* stimuli, namely that of different presentation modes, differing in tempo/rhythm but matched in tonal structure, to study music and emotion. They found that when the same *raga* was presented in distinct presentation modes participants reported elicited emotions with varying levels of arousal. They also found that specific tonal combinations emerged as reliable predictors of emotions that participants reported feeling. These findings indicated that the *ragas* of NICM not only served as interesting and useful acoustic stimuli that could be exploited to study emotion, but also that the structure of the ragas permitted a systematic, controlled investigation of the role of specific features, namely tonality and rhythm in modulating emotions felt by listeners.

KEY CONCEPT 1 **Raga**

Modal melodies comprising the canon of North Indian Classical Music. Each raga is constructed from five or more musical notes, organized into one ascending sequence, and one descending sequence of notes, which together comprise a single melodic framework. Performance of a raga is restricted within the note sequences of its ascending and descending halves, but is improvised in all other respects (e.g., timing between notes; sustain, attack of each note).

In this review we expand upon these findings, and make the case that NICM is tailor-made for disentangling tonal and temporal influences on musical emotion, and thus an invaluable stimulus tool worth bringing to the attention of researchers in all cultural contexts. Specifically, we will build evidence to support that NICM provides (1) a catalog of systematically varying emotion valence, best reflected in the **Circle of** ***Thaats*** (described below); and (2) a form of musical stimulus which has embedded in its very structure an experimentally controlled manipulation of rhythm and tempo keeping tonality constant, allowing for the disentanglement of tonal from rhythmic influences on emotion.

KEY CONCEPT 2 **Circle of *Thaats***

The Circle of *Thaats* organizes the ten canonical *thaats* into a system of incremental variation in tonal ratio (#minor/#Major), with clockwise movement adding Major intervals, and counterclockwise movement subtracting minor intervals. Our lab has previously demonstrated the correlation between emotional valence and tonal ratio in Mathur et al. (2015). For researchers, the Circle can be used as a “dial” for be systematically and gradually manipulating valence.

The review is organized as follows: We begin with the concepts of consonance and dissonance, one of the primary means by which subjective impressions and emotional responses to music arise predictably from frequency ratios between different notes. We then provide an overview of the NICM system, in which different combinations of consonances and dissonances, in the form of tonal intervals, comprise a canon of melodic themes, as the aforementioned *ragas*, with prescribed emotional functions. We then segue to an overview of Mathur et al. (2015), which showed that distinct emotional experiences are reported by listeners for each *raga*, and expand upon these findings by demonstrating that these inter-*raga* emotion differences vary systematically and predictably as a function of minor-to-major tonal interval ratios. Finally, we generalize the findings of Mathur et al. to argue that the structure of NICM is well-positioned for empirical studies of the subtleties and universality of emotions communicated through sound.

On a final introductory note, throughout the discussion that follows we refer to musical emotions as being elicited, induced, etc. in listeners, as opposed to using terms like perceived or identified. This choice of terminology is intentional, as the study by Mathur et al. (2015) which motivated this review was explicitly in the latter camp of the debate between cognitivists and emotivists. The cognitivist view is that listeners do not actually feel emotions when they listen to music, they *perceive* the emotions being expressed (Kivy, 1989). Emotivists, on the other hand, argue that music truly induces emotions, such that a happy tune elicits the same autonomic nervous system responses as any other happy experience (Scherer and Zentner, 2001; Sloboda and Juslin, 2010). For a complete overview of how music elicits emotion, see Juslin and Västfjäll (2008), who provide an extensive review and model for what they argue are the six mechanisms by which music induces emotion: brain stem reflexes, conditioning, visual imagery, contagion, episodic memory, and expectancies fulfilled or denied.

## Consonance, dissonance, and tonal intervals—from quantitative sound qualities, to qualitative musical impressions

Once a musical note leaves an instrument or vocal tract, its timbre, and pitch produce minute fluctuations in air pressure around the listener, triggering electrophysiological impulses in the cochlea which then travel through the brain stem and midbrain en route to specialized subregions of the auditory cortex, where they are imbued with emotional interpretation and memory by higher cognitive processes in the orbitofrontal region of the prefrontal cortex (Zatorre, 2005). It is in this way that objective physical changes in an acoustic signal induce psychological effects as subjective and abstract as feelings, turning acoustic features into psychoacoustic phenomena (Juslin, 1997; Laukka et al., 2013). Communication of the intended emotion, then, depends upon the musician/composer encoding the emotion in acoustic cues, and the listener successfully decoding these acoustic features from psychophysiological stimulation to emotional meaning. Of the various acoustic cues embedded in music, consonance is the most frequently cited as central to influencing emotion perception. Subjectively speaking, consonance and dissonance describe a level of sweetness/harshness of the sound (Zentner and Kagan, 1998). In terms of the aforementioned encoding/decoding communication between composer and listener, consonance encodes a sense of resolution into a composition, dissonance a sense of unresolved tension (Limb, 2006). Indeed, Kamien (2008, p. 41) describes consonance and dissonance qualitatively stating that “A stable tone combination is a consonance; consonances are points of arrival, rest, and resolution. An unstable tone combination is a dissonance; its tension demands an onward motion to a stable chord. Thus dissonant chords are “active;” traditionally they have been considered harsh and have expressed pain, grief, and conflict.”

Studies have confirmed the presence of an innate preference for consonance over dissonance, even in infant populations (Schellenberg and Trehub, 1996; Juslin and Zentner, 2002). Early investigations of the human auditory system revealed that the human ear can disentangle the harmonic overtones of a series if they are separated by a *critical bandwidth* (Plomp and Mimpen, 1968). The ability of the auditory nerve fibers to resolve closely spaced frequencies, then, leads to subjective impressions of pleasant sounds or consonance, whereas the inability to clearly resolve closely spaced frequencies resulted in the impression of dissonance or a “harsher” sound (Von Helmholtz, 1912; Plomp and Levelt, 1965).

Musically speaking, the range of consonance and dissonance that result from different bandwidths is determined by *tonal intervals* (Plomp and Levelt, 1965). The tonal interval is determined by two tones, one of which is conventionally the *tonic* (Parncutt and Hair, 2011). The *tonic* is the root note around which a musical piece is organized, providing a reference for each tone that is sounded during the performance. Tonal intervals produce impressions of consonance if the frequency differences exceed the critical bandwidth (Plomp and Levelt, 1965).

In Parncutt and Hair's (2011) deconstruction of consonance and dissonance, they argue that the two are not in fact diametrically opposed musical phenomena, as they arise from different relationships between tones in a piece of music, some of which are “vertical,” others “horizontal,” in terms of their placement in staff notation. Simultaneously played tones (e.g., as in a chord) have a vertical relationship, whereas the tonal differences between notes or chords separated temporally (e.g., as in a melody) have a horizontal relationship (p. 139): “In a holistic approach, consonance can be promoted by spectral harmonicity (vertical), harmonic proximity or pitch commonality (horizontal), and familiarity (both vertical and horizontal); dissonance by roughness (vertical) and linear pitch distance (horizontal).” Whilst this less dichotomous definition of consonance and dissonance is somewhat specific to Western music (NICM does not rely on the harmonic progressions of chords, as the tanpura drone serves the purpose of providing the tonic root from which tension/resolution are implied), it does suggest that gauging the consonance/dissonance of NICM ragas is a function of both the relationships between the notes, or *swaras*, of a raga and the tonic drone, and the relationships between the *swaras* of a raga in the melodic, horizontal sense.

Tonal intervals form an important organizational principle of musical systems (Castellano and Krumhansl, 1984). When assembled in different combinations, they form diatonic musical modes, the basis of melody construction in any musical system. Due to varying ecological settings, resources, and instrument constructions, some modes used by different musical systems are unique to their native culture (Perlovsky, 2010). Most of our current understanding of the emotions associated with music—emotions presumed to be universal—has come from studies using the modes of Western classical music. Though the literature has consistently shown that listeners associate major and minor modes with positive and plaintive emotions, respectively (Gagnon and Peretz, 2003), increasing work in the field of ethnomusicology suggests that different tonal systems may be able to elicit a subtler gradation of emotions (Thompson and Balkwill, 2010).

## North indian classical music

The two dominant genres of Indian music are North Indian *Hindustani* classical music, and South Indian *Carnatic* classical music. Whilst the styles of singing, presentation of the notes, emphasis on structure of the musical modes and instruments used in each vary, Hindustani and Carnatic music share many common features, from the *raga* system, to the use of *gamakas* (similar to *vibrato)* and *portamento* (phrase-leading accents of rapidly increasing pitch; Capwell, 1986; Swift, 1990). That the arguments made below are made with respect to NICM is due to the fact that the Mathur et al. study from which the data were drawn focused on NICM *ragas*; we suspect the same to hold true for Carnatic music as well.

### Thaats

**Figure 1:**
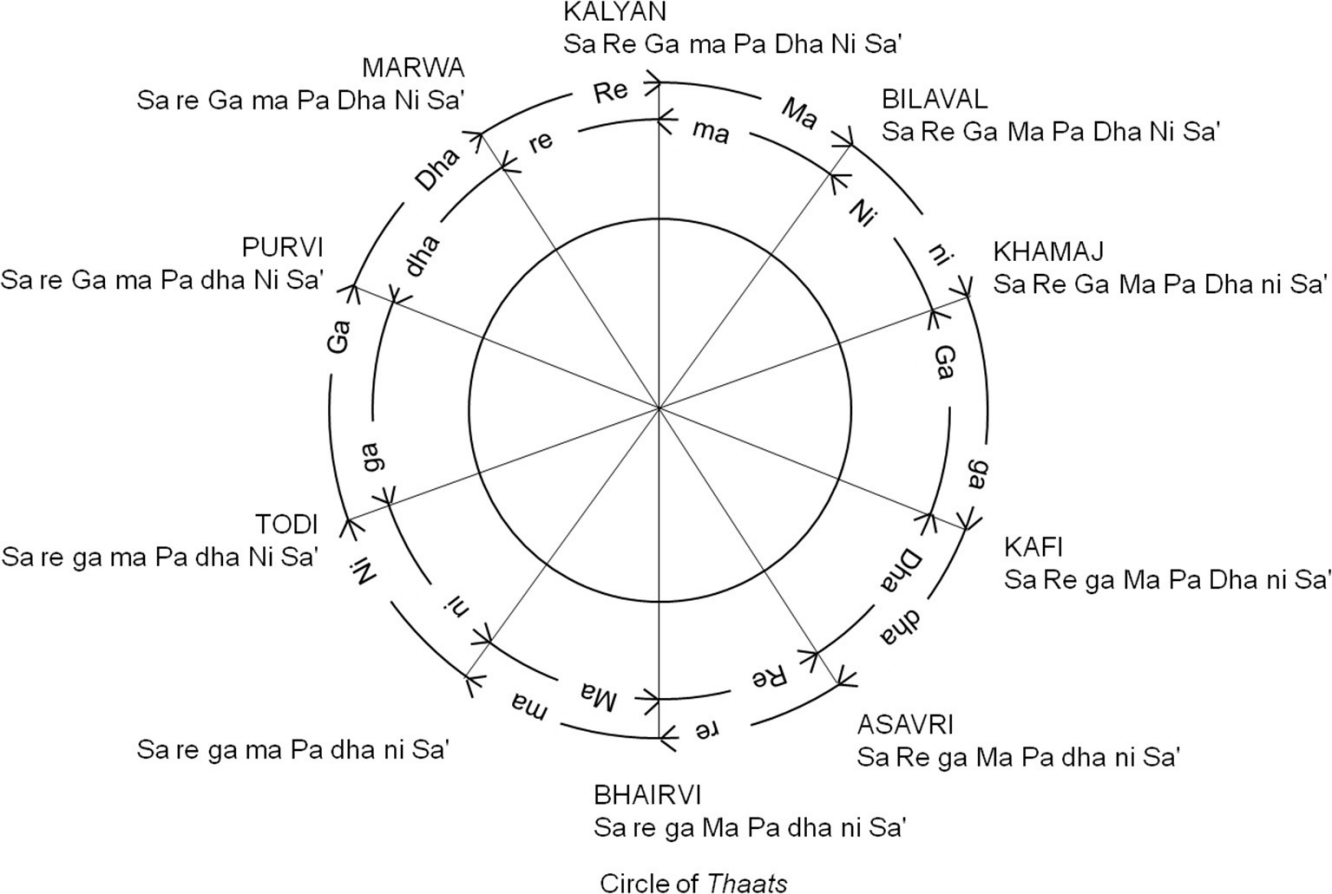
Circle of *Thaats*. The circle of *thaats* illustrating inter-*thaat* distance. Nine of the ten *thaats* on the circle are members of Bhatkhande's classification system (Bhatkhande, 1934). No. 7 *thaat* Bhairav is not currently used in NICM. *Thaat Bhairav* with the scale of *S r G M P d N* is not represented within the circle of *thaats* (Adapted from Jairazbhoy, 1995, p. 59).

The canon of standard NICM *ragas* is categorized and organized around a series of heptatonic scales known as *thaats* (Figure 1; Jairazbhoy, 1995). In the most widely accepted NICM system, there are 10 *thaats* consisting of different sequential combinations of 12 notes. Similar to Western Classical Music, the basic set of tones and tonal intervals used in NICM are the 12-tone octave divisions (Castellano and Krumhansl, 1984; Bowling et al., 2012). While Western music is based on tones with defined frequencies (e.g., A = 440 Hz) NICM music is constructed from tonal intervals, known as *swaras*, which are defined in relation to a tonic tone (in practice this tonic takes the form of a drone note, described below). The “major” intervals (i.e., natural notes) are the *shuddh swaras* while the “minor” intervals are the *komal swaras*. The tonal intervals are *Sa, Re, Ga, Ma, Pa, Dha*, and *Ni*, either in their *shush* (major) or *komal* (minor) form, but never both within the same ***thaat*** (Bhatkhande, 1934).

KEY CONCEPT 3 **Thaat**

The ten heptatonic scale families which are used to classify the canon of North Indian Classical ragas into tonally similar groups. Not all ragas in a given *thaat* include every note of that parent *thaat*, but all ragas in a *thaat* can be derived from its defining scale.

The fourth natural note, *Ma shuddh*, has a variant known as the *tivr* or the augmented fourth (raised by a semitone). Table 1 provides a reference to the *shuddh (major)* and *komal (minor)* intervals, and their Western equivalences, for readers versed in Western music theory.

**Table 1:** Indian/western tonal interval equivalences and frequency ratios.

**Interval name**	**Abbreviations**	**Tonal interval (Western scale)**	**Frequency ratio**
Shadja	Sa	Perfect Unison (tonic)	1
Komal Rhishabha	Re	Minor second	16/15
Shuddha Rhishabha	Re	Major second	10/9
Komal Gandhara	Ga	Minor third	6/5
Shuddha Gandhara	Ga	Major third	5/4
Madhyama	Ma	Perfect fourth	4/3
Tivra Madhyama	Ma	Tritone	45/32
Panchama	Pa	Perfect fifth	3/2
Komal Dhaivata	Dha	Minor sixth	8/5
Shuddha Dhaivata	Dha	Major sixth	5/3
Komal Nishada	Ni	Minor seventh	9/5
Shuddha Nishada	Ni	Major seventh	15/8
Shadja	Sa'	Perfect Octave	2

Across all *thaats* however, the tonic (*Sa*) and fifth (*Pa*) are considered immutable. Thus theoretically, there can be 32 different *thaats*, but Bhatkhande's early census and classification of traditional *ragas* found that the vast majority of *ragas* can be categorized into 10 families or “*thaats*.” The prominent *thaats* have their names adopted from eminent *ragas* that are derived of the same mode though the *ragas* themselves need not necessarily be heptatonic.

It is believed that the most common modes were chosen by Bhatkande such that the structure and practicality were both preserved (Chordia et al., 2008). These 10 canonical *thaats*, and the relationships between them, are summarized by the Circle of *Thaats* (Figure 1, adapted from Jairazbhoy, p. 59). As seen in Figure 1, since all *thaats* are heptatonic, as one moves along the circle starting from *Bilawal* (which has only major notes) in a clockwise manner, the number of minor intervals systematically increases till one reaches a *thaat* that has no name (but has the highest number of minor intervals). Thus *Bilawal* and the *thaat* with no name are diametrically opposite each other. Continuing further, as one move from *Todi*, the number of minor intervals decreases, and the number of major intervals increases systematically till one reaches *Bilawal* again.

### Ragas

*Thaats* provide a useful classification framework, but the core of NICM is the *raga* (Bhatkhande, 1934; Vatsyayan, 1996) The word “*raga*,” which originated in Sanskrit, is defined as “the act of coloring or dyeing;” in this case, the mind and its emotions. The *raga* was thus conceived as a modal melody capable of eliciting specific emotions, or *rasas*.

An intrinsic difference between Indian classical and Western classical music is the *tonic* drone, usually played by a *tanpura*, provides a reference to the listener (a tonic *Sa*, often accompanied by a fifth *Pa* and/or octave *Sa*') creating tonal relationships with the “solitary” melody line of the performance. Because the drone is sounded throughout the presentation of the *raga*, the entire piece can be viewed as a presentation of intervals, not just between notes of the melody line, but between each note and the *Sa* drone. Table 1 dictates the 12 *swaras* of *Hindustani* music displaying their Western classical counterparts, and the frequency ratio of the given note to the tonic.

#### Tonal composition

Each *raga* uses a set of five or more notes from the seven comprising its parent *thaat* to construct a melody. Multiple *ragas* are generated from a single*thaat*, each distinguished by its own signature phrase (*pakar*) and a defined frequency of occurrence of particular notes, *vadi* being the most prominent note and *samvadi* being the second most prominent (Jairazbhoy, 1995; Mathur et al., 2015). This feature allows two *ragas* to have the exact same note selection, yet sound different due to varying emphasis on the notes. *Bhupali*, belonging to *thaat Kalyan*, and *Deskar* to *thaat Bilawal*, are pentatonic *ragas* and use the notes that are common to both *thaats* (Sadhana, 2011). Therefore, even while casually interchanged at times, it is important to understand that a *raga* is not synonymous with a scale; it is a modal melody comprising a defined note selection, differentiated not only on the basis of the notes contained, but also by the frequency of usage of certain notes, the sequencing of ascending (*aarohan*) and descending (*avrohan*) segments, and the *pakar* (Kaufmann, 1965; Leifer, 1987; Jairazbhoy, 1995).

The specific combination of tonal intervals in a *raga* thus create a consonance-dissonance map that then determine which *raga* will feel pleasant on the ear, and which would fall into areas of dissonance, leading to a harsher sound and the need to be resolved into a consonant interval (Helmholtz, 1875; Zuckerkandl, 1956). This subtle combination of tonal intervals permits subtle differences in emotions elicited through music to be investigated using NICM.

#### Rhythmic structure

*Ragas* are usually presented in two consequent sections, the *alaap and the gat*. The *alaap* is an elaborate rendition of the various notes of a *raga*, rendered in free time, introducing and developing the melodic framework, defining characteristics, and mood of a *raga*. The *gat* follows the *alaap*, shifting emphasis to faster sequences of notes, with the accompanying *tabla* (the main NICM percussion instrument) providing a more explicit rhythmic structure while leaving behind most of the subtleties of pitch articulation. Importantly, the tonal structure of the *raga* is consistent between ***alaap and gat***, only the tempo is changed. In this way, the *raga* structure offers an ideal experimental stimulus for disentangling the effects of tempo and tonality: tonality is controlled for between *alaap* and *gat*, while rhythm and tempo are manipulated. It is for this reason that NICM was used by Mathur et al. (2015), enabling the group to isolate the effect of rhythm on emotional elicitationsand, in doing so, demonstrating the unique utility NICM offers as an experimental stimulus.

KEY CONCEPT 4 **Alaap and Gat**

Raga performance has two stages, alaap and gat. The alaap introduces the raga, laying out a tonal framework. The gat introduces the rhythmic accompaniment, increasing in tempo and becoming stricter in rhythmic structure until there is very little room left for improvisation. As both stages use the same scale, changing tempo, ragas are experimental stimuli by nature, with which melodic and temporal effects on emotion can be distinguished.

#### Cultural relationship between *Raga, Rasa*, and *Bhava* (modal melody, mood, and emotion label)

On a more subjective level, emotional intent is a distinguishing feature of the NICM *raga* system. Whereas emotions and moods are implied characteristics of Western Classical music, Indian *ragas* have prescribed emotional effects, or *rasa*s (Vatsyayan, 1996), each *rasa* intended to alter the mood (*bhava*) of the listener in a particular manner. Erotic love (*sringara*), patheticness (*karuna*), devotion (*bhakti*), comedy (*hasya*), horror (*bhayanaka*), repugnancy (*bibhatsa*), heroism (*vira*), fantastical, furious (*roudra*), and peaceful (*shanta*) were named in Bhatkhande's description of *rasa* and *bhava* (Bhatkhande, 1934; Bowling et al., 2012).

Knowing the *bhava* that the *rasa* of a particular *raga* is meant to induce (Mathur et al., 2015), such stimuli are invaluable to musical emotion studies, cross-cultural or otherwise, as the Circle of *Thaats*, and the canon of standard ragas it encompasses, can be utilized as a catalog for eliciting subtle gradations in emotional effect, some of which are culturally universal, others less so. Whilst Western listeners perceive the same basic emotions—happy, sad, angry, disgusted, surprised, fearful—as native listeners in *Hindustani* music, more subtle emotional gradations of basic emotions (e.g., “peacefulness” rather than happiness) are more easily identified by native listeners (Balkwill and Thompson, 1999; William Forde Balkwill et al., 2004; Fritz et al., 2009; Laukka et al., 2013).

## Mathur et al. (2015)

Recently, Mathur et al. (2015) tested the hypothesis that *ragas* elicit distinct emotional feelings. Using 3-min compositions of 12 *ragas*, presented in the form of an online survey, participants rated these *ragas* on the degree to which they elicited different emotions. All *ragas* were composed by a professional musician and rendered on *sarod*, an Indian stringed instrument.

As indicated earlier, Mathur et al. exploited the structure of a raga composition and presented each of the 12 *ragas* in both *alaap* and *gat*. Participants were instructed to rate each excerpt on eight distinct emotions on a 0–4 Likert scale (with 0 being “not at all felt” to 4 being “felt the most”) for each of the following emotion labels: happy, romantic, devotional, calm/soothed, angry, longing/yearning, tensed/restless, and sad. The study did not use a forced choice task but instead sought each *raga* to be rated for each of the eight emotions, sensitive to the fact that a single musical composition can elicit multiple moods. Specifically the study sought to determine if participant responses (1) differed between the emotions experienced by *alaap* and *gat* for various ragas (2) whether the psychophysical variables of rhythm and tonality influenced the emotions experienced.

The first finding of Mathur et al.'s study was that distinct ragas are associated with distinct emotional elicitations. This was similar to the findings reported by Balkwill and Thompson (1999) which showed that even western listeners who were unfamiliar with the tonal system of NICM perceived the intended emotion in *ragas*. However Balkwill and Thompson used ragas only in the *alaap* mode and implemented forced choice task. Participants in that study were required to indicate which of the four target emotions was dominant for the raga. Mathur et al. on the other hand asked each participant to rate the extent to which each of the emotions were *experienced* during the listening of the *raga*. Mathur et al. also found that that when the raga was presented in “*alaap*,” participants ratings were either calm (positive) or sad (negative) emotion. However, when presented in the *gat* condition, a finer discrimination of emotions were elicited (happy, romantic, calm) and (sad, longing, tension). This was the first experimental verification of the hypothesis that distinct emotions are associated with *alaap* and *gat* of a *raga*. Further, since Mathur et al. also used acoustic analysis to extract estimates of rhythmic regularity and tempo, they correlated acoustic features with behavioral ratings of emotional elicitation and were able to demonstrate that high arousal emotions like happy/tensed were associated with *gat*. As elaborated earlier the *gat* follows faster sequences of notes and provides an explicit rhythmic structure. A comparison of these results with those from the study conducted by Balkwill and Thompson showed that tempo and melodic complexity had some predictive power. However this was found only for some differences. Balkwill and Thompson (1999) used psychophysical ratings of tempo and melodic complexity and found that a combination of the two, predicted emotions primarily joy and sadness. Similar results were also reported by Gabrielsson and Juslin (1996) who showed that faster tempo were associated with positive emotions while slower tempo with negative emotions.

What was novel in Mathur et al.'s study was the finding that there is a change in the level of arousal between *alaap* and *gat* for the same raga. Since the tonal structure of the *raga* is preserved between *alaap* and *gat*, only the tempo is changed and the finding that high arousal emotions are associated with gat points to the fact that the *raga* structure is an optimal stimulus to dissociate the effects of tempo and tonality: tonality is controlled for between *alaap* and *gat*, while rhythm and tempo are manipulated. This result from Mathur et al. (2015), illustrated that this unique structure of NICM that enables the isolation of the effect of rhythm on emotion elicitationrenders it as a useful experimental stimulus.

**Figure 2:**
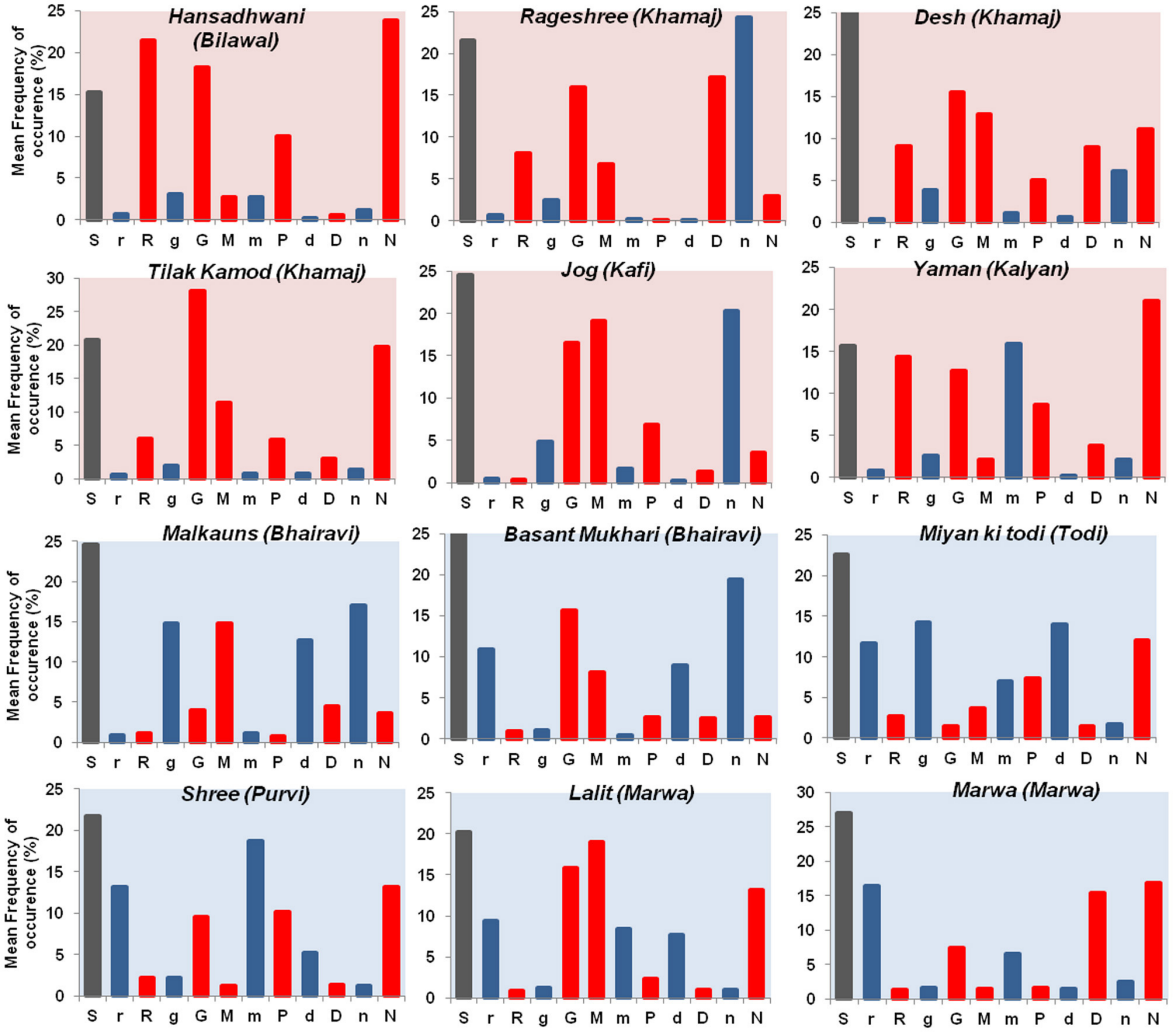
Tonality for *ragas*. The above figure represents the tonic interval distribution for the 12 *ragas* used in the study (Mathur et al., 2015). The tonal distribution of *ragas* rated as “calm” is represented with red background color panel whereas the tonal distribution of *ragas* rated as “sad” is represented with blue background color panel. Within each panel the mean frequency of occurrence of *shuddh swaras* [S (*Sa*), R (*Re*), G (*Ga*), M (*Ma*), P (*Pa*), D (*Dha*), N (*Ni*)] is depicted with red bars whereas the mean frequency of occurrence of *komal swaras* [r(*re*), g(*ga*), m(*ma*), d(*dha*), n(*ni*)] is depicted with blue bars.

Of greater interest was the second primary finding of Mathur et al. (2015) study which showed that specific tonic intervals were robust predictors of elicited emotions. Major intervals were found to be associated with positive emotions and minor intervals to be associated with negative emotions. An analysis of tonal intervals of *ragas*, revealed that *ragas* rated as positive (such as “calm” and “happy”) had a greater mean frequency of occurrence of major intervals (*shuddh swaras*) whereas *ragas* with negative emotion (e.g., sad or tensed) were characterized by an increased frequency of minor intervals (*Komal swaras*). Figure 2 shows a distribution of mean frequency of occurrence of tonic intervals for the 12 *ragas* used in the study. Red bars represent the mean frequency of occurrence of *shuddh swaras* whereas that of *komal swaras* is represented with blue bars for each *raga*. To the best of our knowledge this finding for ragas is novel and has not been reported earlier.

To further explore the findings from Mathur et al. and assess the degree to which emotion ratings agree with *rasa* variation around the Circle of *Thaats*, we first associated a valence score with each *raga*, which is a difference in the ratings of the two highest experienced emotions, calm and sad. As a consequence, a value >0 is associated with positive valence whereas, a difference < 0 is associated with negative valence. Next, we define a tonal ratio, which is the ratio of the number of minor intervals (m) to major intervals (M) for each *raga*. The valence score and tonal ratios estimated are listed in Table 2.

**Table 2:** Ratios of minor (m) to major (M) intervals along with mean ratings for *ragas* belonging to each *thaat* as estimated in Mathur et al. (2015).

***That***	***Scale***	***Ragas* used by Mathur et al. (2015)**	**m**	**M**	**m/M ratio**	**C**	**S**	**C-S**
Bilawal	Sa Re Ga Ma Pa Dha Ni Sa'	Hansadhwani	0	7	0.00	2.32	1.09	1.22
Kalyan	Sa Re Ga ma Pa Dha Ni Sa'	Yaman	1	6	0.17	1.99	1.38	0.61
Marwa	Sa re Ga ma Pa Dha Ni Sa'	Marwa	2	5	0.40	1.63	1.99	−0.37
		Lalit				1.66	2.12	−0.46
Purvi	Sa re Ga ma Pa dha Ni Sa'	Shree	3	4	0.75	1.61	1.87	−0.26
Todi	Sa re ga ma Pa dha Ni Sa'	Miya Ki Todi	4	3	1.33	1.6	1.93	−0.33
No name	Sa re ga ma Pa dha ni Sa'		5	2	2.50			
Bhairavi	Sa re ga Ma Pa dha ni Sa'	Malkauns,	4	3	1.33	2.02	1.56	0.46
		Basant Mukhari				1.75	2.1	−0.35
Asavari	Sa Re ga Ma Pa dha ni Sa'		3	4	0.75			
Kafi	Sa Re ga Ma Pa dha ni Sa'	Jog	2	4	0.50	1.83	1.33	0.5
Khamaj	Sa Re Ga Ma Pa Dha ni Sa'	Tilak Kamod	1	6	0.17	2.31	1.03	1.28
		Ragashree				2.05	1.32	0.73
		Desh				2.21	1.14	1.07

The mean of ratings (by N = 193–206 culturally familiar Indian participants) for “Calm” and “Sad” emotion are represented under the column heading “C” and “S,” respectively whereas the mean difference estimated between calm and sad ratings is given under the column heading “C-S.”

**Figure 3:**
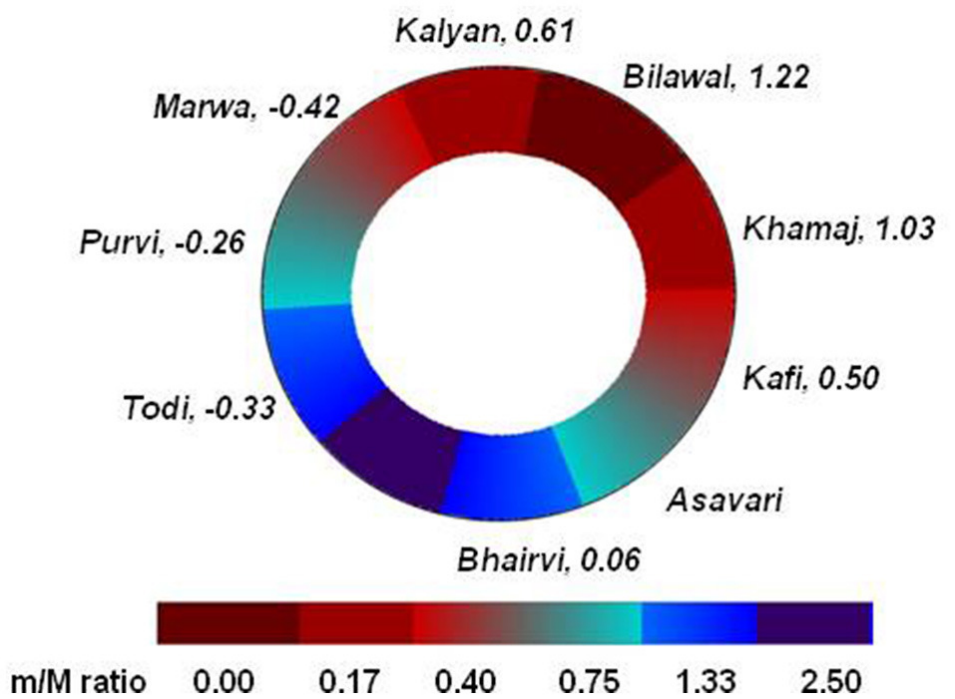
Similarities between the tonal ratio (m/M) and valence of *ragas* when arranged along Circle of *Thaats*. The tonal ratio is color coded in increasing order on a scale of red to blue. Valence values associated with each *thaat* are indicated next to the names of the *thaat*, with negative valence preceded by the minus sign. For *thaats* where multiple *ragas* were included in Mathur et al. a mean valence was estimated based on the ratings in Table 2.

In Figure 3, we compare the **tonal ratios** with the valence score of the *ragas* used by Mathur et al. The tonal ratios and valence score as estimated for various *ragas* are represented along the Circle of *Thaats* (refer to Table 2 and Figure 3). The *thaats* for which emotional elicitations for more than one *raga* were available, an average valence score has been estimated (Table 2). The tonal ratios are expressed on a color scale (red to blue) while the average valence score associated with each *thaat* is indicated along with the name of the respective *thaat*. Figure 3 reveals that *ragas* belonging to *thaat Bilawal* (tonal ratio 0.00), elicits emotions with positive valence (e.g., valence score of *raga Hansadhwani* and *Tilak Kamod* are 1.22 and 1.28, respectively) where *ragas* belonging to *thaat Todi* (tonal ratio 0.57) and *thaat Marwa* (tonal ratio 0.29) evokes emotions of negative valence (e.g., valence score of *Basant Mukhari or Mivan ki Todi* is −0.35 and −0.33, respectively). Thus as the tonal ratio systematically increases moving clockwise from 0 and subsequently decreases, valence follows suit. In effect, the qualitative *rasa* variation adumbrated by the Circle of *Thaats* aligns with quantitative variations in both emotion rating valence and tonal ratio for the ragas tested.

KEY CONCEPT 5 **TONAL RATIO**

The ratio of #minor/#Major intervals in a raga. As minor intervals are dissonant and Major intervals are consonant, this ratio gauges consonance/dissonance across an entire raga scale. Raga tonal ratios align with valences of participants' subjective ratings, suggesting the canon of North Indian ragas is a source for experimental stimuli with for those seeking a range and gradation of valences.

Finally, a third finding of Mathur et al. (2015) was that out of the 12 tonic intervals, the minor second interval (*komal re*) was the best predictor of negative valence. As seen in Figure 3, there are two locations along the circle of *thaats*, which we refer to as transition *thaats* where a change in tonal ratio is accompanied by a change in valence category (refer to Figure 3 and Table 2). These are *Kafi* to *Asavari* (positive to negative valence) and *Purvi* to *Khamaaj* (negative to positive). While symmetrically located on the circle of *thaats*, and similar tonal ratios the subsequent valence associated with the transition *thaats* is quite different. We attribute this to the specific minor intervals involved.

While the minor intervals present in transition *thaats Kafi* to *Asavari* are minor third (*komal ga*) and minor seventh (*komal ni*) those present in transition *thaats Purvi* to *Marwa* are minor second (*komal re*) and tritone (*tivra ma*) respectively. We suggest that the presence of the minor second in the transition *thaats Purvi* and *Marwa* leads to their association with higher negative valence score (−0.26 and −0.42, respectively) as compared to *thaat Kafi* (0.50). While the study by Mathur et al. did not include a *raga* from *thaat Asvari* the results encourage us to hypothesize that the minor second serves a crucial role in conveying negative valence. Further studies should attempt to investigate its role in detail by sampling a larger representation of *ragas* from each *thaat*.

## Conclusion

The purpose of this review was to demonstrate NICM *ragas* as robust stimuli capable of eliciting distinct, predictable emotions, with tonal relationships and rhythmic tempo influencing the valence and strength of emotional effects in the listener. The *ragas* used in Mathur et al. (2015) were only 12 in number, but since they had been sampled across almost all *thaats* we attempted to speculate how the structure of the tonic intervals might predict the emotional valence associated with a *raga*.

Moving around the Circle of *Thaats*, emotional valence systematically varied along with the tonal ratios of each *thaat*. In this way, music emotion researchers may find experimental utility in the Circle of Thaats, as a catalog of stimuli varying in degrees of valence not only systematically, but *incrementally*, in the sense of finer gradations of valence than the more binary notions of “positive”/”negative” “happy”/”sad” typically ascribed to consonance and dissonance effects on emotion. In addition, built into the very structure of Indian compositions is an experimental manipulation of rhythmic tempo between *alaap* and *gat*, keeping tonal intervals constant, which in Mathur et al. (2015) revealed that the musical differences between sadness and tension, calmness and happiness may be more a function of rhythm than melody.

In sum, the catalog of systematically and incrementally varying emotional valence comprising the Circle of *Thaats*; and a varying rhythmic structure which controls for tonality across a single *raga*, together make NICM music an invaluable auditory stimulus, tailor made and uniquely useful for experimentally controlled studies of musical emotion. We acknowledge that at present the preliminary results discussed here are speculative and require more detailed investigation. Since the tonic ratio is directly related to emotional response, further studies should also probe the nature of this relationship in influencing the strength of arousal of positive or negative valence of a *raga*, a feature that is often adopted by various performing artistes that has not been experimentally investigated. Future research would also do well to test the degree to which the constant tonic drone amplifies the strength of the emotional valences induced via the consonances and dissonances of these tonic ratios.

Finally, whilst a main aim of this review was to describe why *ragas* are a uniquely useful experimental stimuli for studies of music and emotion, this methodological prescription comes with an important caveat. *Ragas* are musical stimuli with deep, specific cultural origins and associations (Wieczorkowska et al., 2010). But although they have been shown to elicit culturally-specific emotions which appear to be lost on non-native listeners, they also convey emotions that are shared between native and non-native listeners (Laukka et al., 2013). Consequently, for experiments using *raga* stimuli for cross-cultural research this is crucial to note, as beyond universal emotions there are enculturated emotions elicited by culture-specific cues in music. For studies using only Western or only Indian samples, however, such cultural effects should not be a concern, as all participants would be equally advantaged or disadvantaged in identifying culturally dependent musical cues.

On a final, related point, it is important to note that the usage of *ragas* in the Western music cognition literature is nearly always in the context of cross-cultural differences. We hope Western readers come away from this review with an understanding that *ragas* can be thought of as more than “world music,” and useful for more than only cross-cultural studies of music cognition and emotion.

Ultimately, we hope this review brings the unique experimental value of NICM to the attention of music emotion researchers, useful for investigating emotional elicitations to music, within, between, and across different cultures.

## Author contributions

NS: designed the study and wrote the paper; JV and JA: contributed to paper writing; AM: collected data, conducted analysis, and contributed in writing the paper.

### Conflict of interest statement

The authors declare that the research was conducted in the absence of any commercial or financial relationships that could be construed as a potential conflict of interest.
